# Activation of matrix metalloproteinases and FoxO3a in HaCaT keratinocytes by radiofrequency electromagnetic field exposure

**DOI:** 10.1038/s41598-021-87263-2

**Published:** 2021-04-07

**Authors:** Ju Hwan Kim, Dong-Jun Kang, Jun-Sang Bae, Jai Hyuen Lee, Sangbong Jeon, Hyung-Do Choi, Nam Kim, Hyung-Gun Kim, Hak Rim Kim

**Affiliations:** 1 Department of Pharmacology, College of Medicine, Dankook University, 119 Dandaero, Cheonan, Chungnam, 31116 Republic of Korea; 2 Medical Laser Research Center, Dankook University, Cheonan, Chungnam, Republic of Korea; 3 Department of Nuclear Medicine, College of Medicine, Dankook University, Chungnam, Republic of Korea; 4 Radio and Broadcasting Technology Laboratory, ETRI, Daejeon, 34129 Republic of Korea; 5 School of Electrical and Computer Engineering, Chungbuk National University, Cheongju, Chungbuk 28644 Republic of Korea

**Keywords:** Cell biology, Physiology, Risk factors

## Abstract

As the skin is the largest body organ and critically serves as a barrier, it is frequently exposed and could be physiologically affected by radiofrequency electromagnetic field (RF-EMF) exposure. In this study, we found that 1760 MHz RF-EMF (4.0 W/kg specific absorption rate for 2 h/day during 4 days) exposure could induce intracellular reactive oxygen species (ROS) production in HaCaT human keratinocytes using 2′,7′-dichlorofluorescin diacetate fluorescent probe analysis. However, cell growth and viability were unaffected by RF-EMF exposure. Since oxidative stress in the skin greatly influences the skin-aging process, we analyzed the skin senescence-related factors activated by ROS generation. Matrix metalloproteinases 1, 3, and 7 (MMP1, MMP3, and MMP7), the main skin wrinkle-related proteins, were significantly increased in HaCaT cells after RF-EMF exposure. Additionally, the gelatinolytic activities of secreted MMP2 and MMP9 were also increased by RF-EMF exposure. FoxO3a (Ser318/321) and ERK1/2 (Thr 202/Tyr 204) phosphorylation levels were significantly increased by RF-EMF exposure. However, Bcl2 and Bax expression levels were not significantly changed, indicating that the apoptotic pathway was not activated in keratinocytes following RF-EMF exposure. In summary, our findings show that exposure to 1760 MHz RF-EMF induces ROS generation, leading to MMP activation and FoxO3a and ERK1/2 phosphorylation. These data suggest that RF-EMF exposure induces cellular senescence of skin cells through ROS induction in HaCaT human keratinocytes.

## Introduction

Owing to the rapid development of modern information and communication technology, humans are constantly exposed to frequent electromagnetic fields. Specifically, public concern about the possible harmful effects of radiofrequency electromagnetic fields (RF-EMF) generated by electronic devices on the human body is continuously increasing. In 2011, the International Agency for Research on Cancer categorized RF-EMF as Group 2B, which is possibly carcinogenic to humans and warned the public about the potential risks^1^.

The skin is the largest body organ and critically serves as a barrier by protecting humans from various external environmental stresses^2^. Therefore, it is frequently exposed to and can be physiologically affected by RF-EMF insults. Notably, protein expression was found to be altered in human skin upon exposure to 900 MHz RF-EMF^3^. It is thus important to study the biological effects of RF-EMF on the skin since body RF-EMF exposure would be delivered to the outermost skin and then penetrate into the skin.

As an outer soft tissue, skin-aging processes are accompanied by phenotypic changes in cutaneous cells with structural and functional changes in extracellular matrix components. Among various factors, oxidative stress in the skin greatly influences the skin-aging processes. Furthermore, skin aging is a crucial issue in biology and is caused by time-dependent internal and extrinsic factors such as ultraviolet (UV) light^2^. Chronic UV exposure to skin induces oxidative stress, which greatly influences the skin-aging process^4^. Oxidative stress in the skin mainly causes reactive oxygen species (ROS) generation, which then activates matrix metalloproteinase (MMP)-mediated aging, inflammation-mediated aging, and apoptosis-mediated aging^5^. Notably, RF-EMFs stimulate ROS generation both in vivo^6,7^ and in vitro^8,9^.

Despite having a pivotal role in protecting the body, how human skin is biologically affected by RF-EMF exposure is not well understood. In this study, we investigated the possible biological responses of human keratinocytes to RF-EMF exposure, including skin-aging processes. Moreover, we describe the possible biological effects of exposing the skin to 1760 MHz RF-EMF (specific absorption rate [SAR], 4.0 W/kg value for 2 h daily for 4 days), including the induction of intracellular ROS and activation of skin senescence factors, in human keratinocytes.

## Results

### Cellular ROS levels are significantly increased in HaCaT cells after RF-EMF exposure

**Figure 1:**
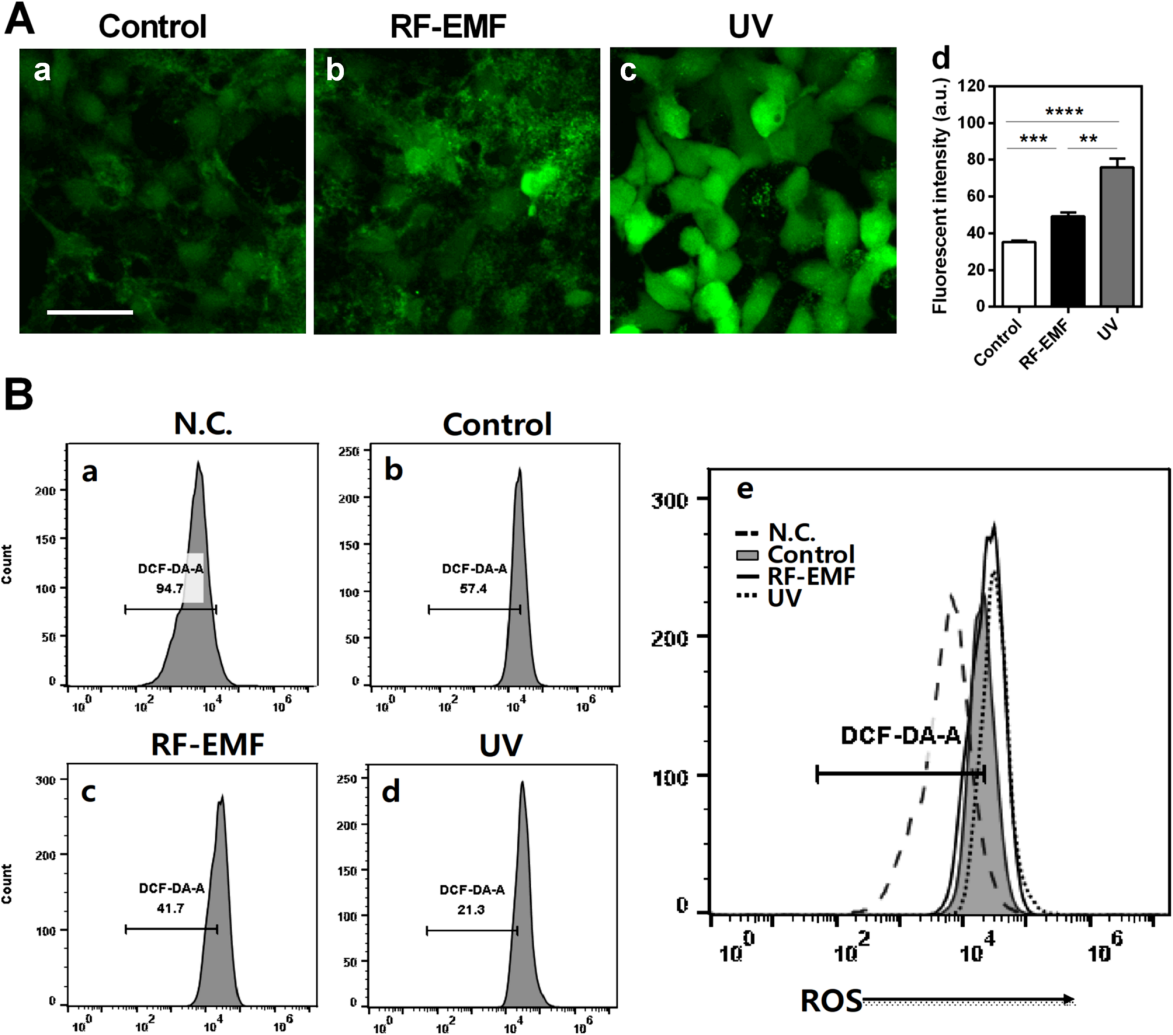
Exposure to radiofrequency electromagnetic field (RF-EMF) increased reactive oxygen species (ROS) production in HaCaT cells. HaCaT cells were exposed to 1760 MHz RF-EMF with 4.0 W/kg, 2 h daily for 4 days. The cells exposed to ultraviolet A (UVA; 365 nm, 6 W for 3 h) were used as a positive control for ROS generation. (**A**) ROS levels were measured using the fluorescent probe 2′,7′-dichlorofluorescin diacetate (DCF-DA). Fluorescence intensities were analyzed using ImageJ. The bars indicate the mean ± SEM. Statistically significant levels were evaluated using two-tailed, unpaired Student’s *t*-tests (**p < 0.01, ***p < 0.001, ****p < 0.0001). (**B**) HaCaT cells were treated with DCF-DA and cellular ROS levels in each condition were analyzed using flow cytometry (BD Accuri C6 and Flow Jo software). Negative control (N.C.); cells were untreated with DCF-DA (n = 3).

To evaluate the possible effects of RF-EMF exposure on skin cells, human keratinocyte HaCaT cells were exposed to1760 MHz RF-EMF at 4.0 W/kg, 2 h daily for 4 days. Since ROS generation in the skin is one of the main causes of skin aging, ROS generation in HaCaT cells induced by RF-EMF exposure was measured using the fluorescent probe 2′,7′-dichlorofluorescin diacetate (DCF-DA). ROS production was strongly induced by UV exposure, and cells exposed to ultraviolet A (UVA) were used as a positive control (Fig. 1). Fluorescence images were obtained using a confocal microscope and then analyzed using the ImageJ program. The results showed that ROS levels were significantly increased by RF-EMF exposure (*p* < 0.001; Fig. 1A). Additionally, we analyzed ROS production levels in whole cells using flow cytometry with Flow Jo software. These data indicated that intracellular ROS production was increased to 15.7% in HaCaT cells after RF-EMF exposure, showing that 58.3% of RF-EMF-exposed cells were stained with DCF (41.7% cells unstained with DCF; Fig. 1B c), whereas 42.6% of the control cells were stained with DCF (57.4% cells unstained with DCF; Fig. 1B b). Additionally, as a positive control, UV-exposed cells strongly generated ROS, and results indicated that 78.7% of the cells were stained with DCF after UV exposure (21.3% cells unstained with DCF; Fig. 1B d). Therefore, these data suggested that cellular levels of ROS production in human keratinocyte HaCaT cells would be significantly increased by 1760 MHz RF-EMF exposure.

### RF-EMF exposure does not affect HaCaT cell morphology, numbers, and viability

**Figure 2:**
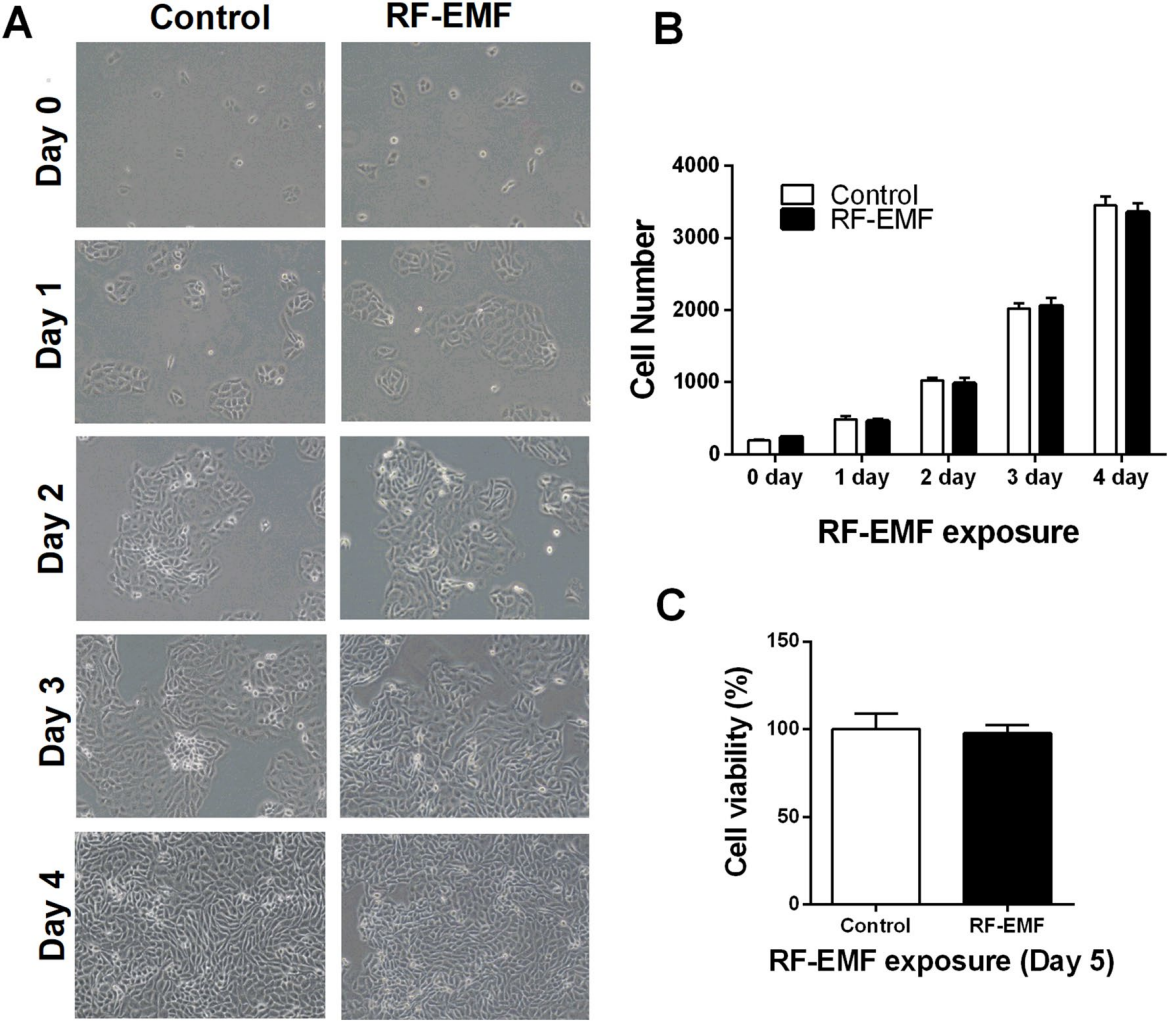
HaCaT cell morphology, number, and viability after radiofrequency electromagnetic field (RF-EMF) exposure. HaCaT cells were exposed to 1760 MHz RF-EMF with 4.0 W/kg, 2 h daily for 4 days. (**A**) Cellular morphologies were considered using microscopy (Olympus CKX53). (**B**) The number of cells was counted using CKX-CCSW software, which provides a record of quantitative data automatically. (**C**) Cell viability was measured on day 5 using tetrazolium salts (WST-1) for the cell proliferation assay (n = 3).

To elucidate the possible effects of exposure to RF-EMF on cell growth, we considered the cell morphology, numbers, and viability in each condition. During exposure to 1760 MHz RF-EMF with 4.0 W/kg for 2 h daily for 4 days, the cell images were captured daily using a microscope. The results showed no significant differences in cell morphology between the control and RF-EMF-exposed cells, indicating that RF-EMF exposure did not affect the normal morphology of HaCaT cells (Fig. 2A). In addition, the cell number was counted automatically using a specific program (CKX-CCSW software) during the aforementioned exposure. HaCaT cell numbers between the control and RF-EMF-exposed conditions were constant between days 0 and day 4 (Fig. 2B), indicating that RF-EMF exposure did not affect cell growth under these conditions. Finally, to evaluate the possible cytotoxicity of RF-EMF, HaCaT cell viability after 4 days of RF-EMF exposure was tested using a WST-1 cell proliferation assay. The viability of HaCaT cells was unaffected by 1760 MHz RF-EMF at 4.0 W/kg for 2 h daily for 4 days (Fig. 2C). These data indicated that the overall growth and viability of HaCaT cells were not strikingly affected by exposure to these RF-EMF conditions, even though ROS production was significantly increased.

### MMP expression levels and activity are significantly increased in HaCaT cells by RF-EMF exposure

**Figure 3:**
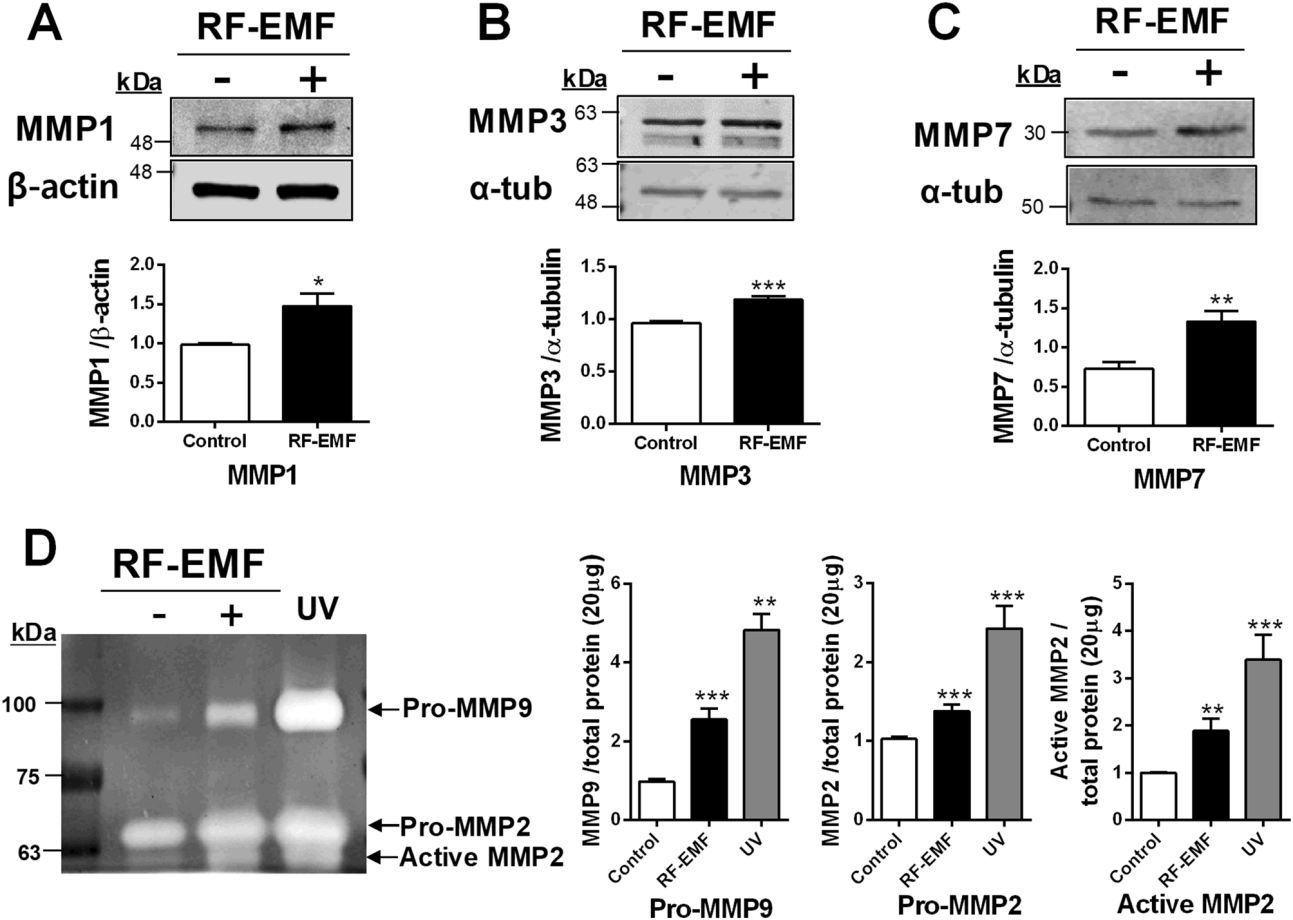
Matrix metalloproteinase (MMP) expression and activity in HaCaT cells after radiofrequency electromagnetic field (RF-EMF) exposure. Representative immunoblot of MMP1 (**A**), MMP3 (**B**), and MMP7 (**C**) in HaCaT cells after 1760 MHz RF-EMF exposure (4.0 W/kg, 2 h/day for 4 days). The levels of MMPs were normalized to those of β-actin or α-tubulin. (**D**) Zymography for gelatinolytic activity of MMP-9 and MMP-2 in HaCaT cells. The cells exposed to ultraviolet A (UVA; 365 nm, 6 W for 45 h) were used as a positive control. The data indicate the mean ± SEM. Levels of statistical significance were evaluated using two-tailed, unpaired Student’s *t*-tests; *p < 0.05, **p < 0.01, ***p < 0.001 vs. control (n = 5).

MMP production induces skin aging. To determine whether MMPs such as MMP1, 3, and 7 would be affected by RF-EMF exposure in human keratinocyte HaCaT cells, we examined their expression and activity levels after this treatment. MMP1 and MMP3 protein expression levels were significantly increased by nearly 50% and 30% following RF-EMF exposure (Fig. 3A,B), respectively. In addition, MMP7 expression was significantly increased to almost double that of the control cells (Fig. 3C). These results indicated that 1760 MHz RF-EMF with 4.0 W/kg for 2 h daily for 4 days could significantly upregulate the expression of these markers in HaCaT cells. Furthermore, we studied MMP2 and MMP9 activities by performing a gelatinolytic activity assay. The secreted gelatinolytic activities of MMP2 and MMP9 were determined using gelatin zymographic analysis of supernatants of HaCaT cell culture in each condition (Fig. 3D). The MMP2 and MMP9 bands were visualized at 72 kDa and 92 kDa, respectively (Fig. 3D). The supernatant from HaCaT cells irradiated with UV for 45 min was used as a positive control. The results showed that pro-MMP2 and pro-MMP9 existed at a much higher level (nearly 2.5-and 1.5-fold, respectively) in RF-EMF-exposed cells compared to that in the control cells (Fig. 3D). Meanwhile, the MMP2 active form appeared at 62 kDa and increased two-fold in RF-EMF-exposed HaCaT cells compared to levels in control cells (Fig. 3D), and activated MMP9 was not detected in this study, indicating that MMP2 and MMP9 gelatinolytic activities were upregulated in HaCaT cells after 1760 MHz RF-EMF exposure.

### The apoptotic pathway is not activated by RF-EMF exposure in HaCaT cells

**Figure 4:**
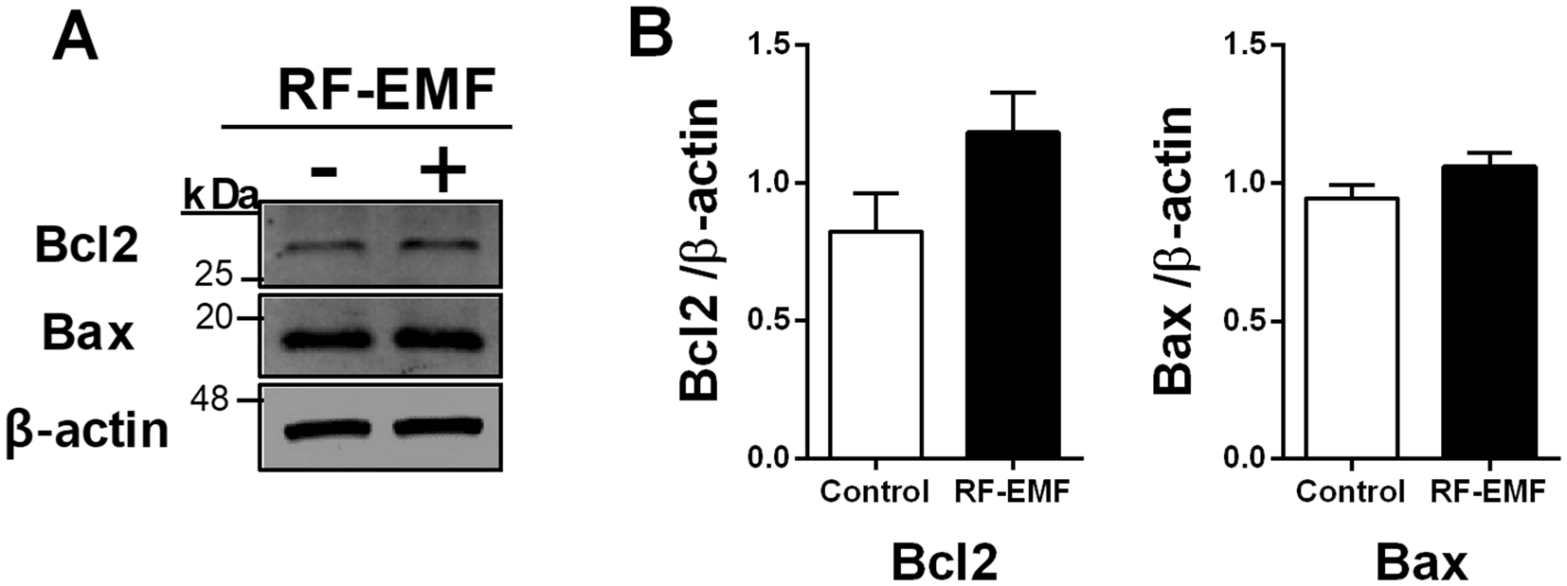
Bcl2 and Bax expression levels in HaCaT cells after radiofrequency electromagnetic field (RF-EMF) exposure. (**A**) Representative immunoblot of Bcl2 and Bax in HaCaT cells after 1760 MHz RF-EMF exposure (4.0 W/kg, 2 h/day for 4 days). (**B**) The expression levels of Bcl2 or Bax were normalized to those of β-actin. The bars indicate the mean ± SEM. Levels of statistical significance were evaluated using two-tailed, unpaired Student’s *t*-tests (n = 3).

To determine whether the apoptotic pathway was activated in keratinocytes after RF-EMF exposure, HaCaT cells were exposed to 1760 MHz RF-EMF at 4.0 W/kg (2 h/day) for 4 days, and then, western blotting was performed with anti-Bcl2 and anti-Bax antibodies. The levels of both Bcl2 (anti-apoptotic factor) and Bax (pro-apoptotic member) did not significantly change in HaCaT cells after RF-EMF exposure (Fig. 4), indicating that this did not activate the apoptotic pathway in human keratinocytes under these experimental conditions.

### RF-EMF exposure induces the activation of MAPK signaling but not the inflammatory pathway in HaCaT cells

**Figure 5:**
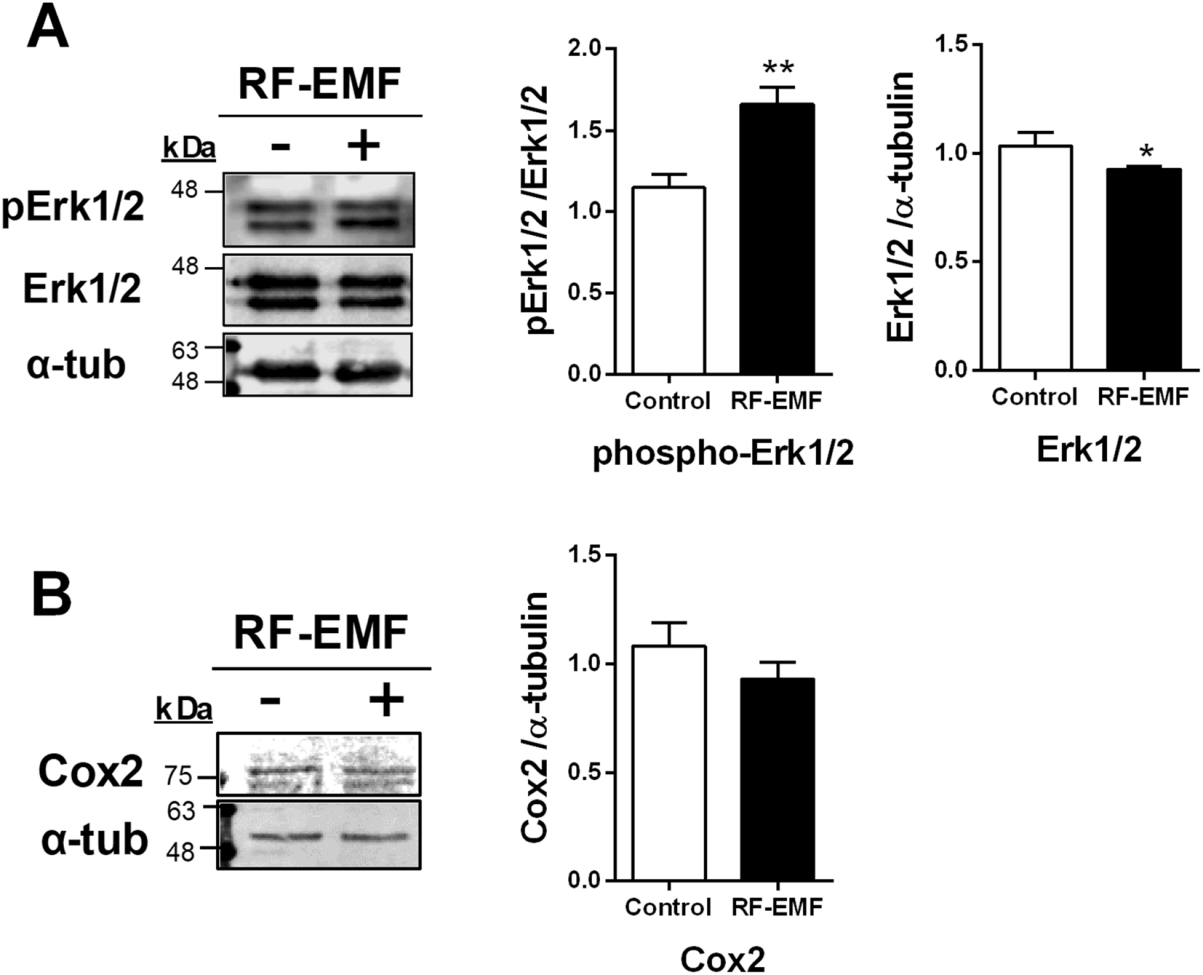
Phosphorylation of ERK1/2 is significantly increased, but Cox2 expression is unaltered, in HaCaT cells after radiofrequency electromagnetic field (RF-EMF) exposure. Representative immunoblot of ERK1/2, phospho-ERK1/2 (Thr202/Tyr 204) (**A**) and Cox2 (**B**) in HaCaT cells after 1760 MHz RF-EMF exposure (4.0 W/kg, 2 h/day for 4 days). The data were normalized to α-tubulin levels. The bars indicate the mean ± SEM. Levels of statistical significance were evaluated using two-tailed, unpaired Student’s *t*-tests; *p < 0.05, **p < 0.01 vs. control (n = 6).

MAPK signaling and inflammatory processes could be activated by increased ROS production in skin cells. To explore whether RF-EMF exposure could induce MAPK pathway activation in human keratinocytes, the expression and activation of extracellular signal-regulated kinase 1/2 **(**ERK1/2) were studied in HaCaT cells after 1760 MHz RF-EMF exposure. Whereas, the ERK1/2 phosphorylation level was significantly increased, its expression level was decreased by RF-EMF exposure (Fig. 5A), suggesting that RF-EMF exposure activates MAPK signaling, and specifically ERK1/2 (Thr 202/Tyr 204), although the total ERK 1/2 expression level was decreased in HaCaT cells. Additionally, we examined whether the inflammatory pathway in human keratinocytes could be induced by 1760 MHz RF-EMF. However, the data showed that cyclooxygenase 2 (Cox2) expression was not induced by RF-EMF exposure in HaCaT cells (Fig. 5B).

### FoxO3a phosphorylation levels are significantly increased in HaCaT cells after RF-EMF exposure

**Figure 6:**
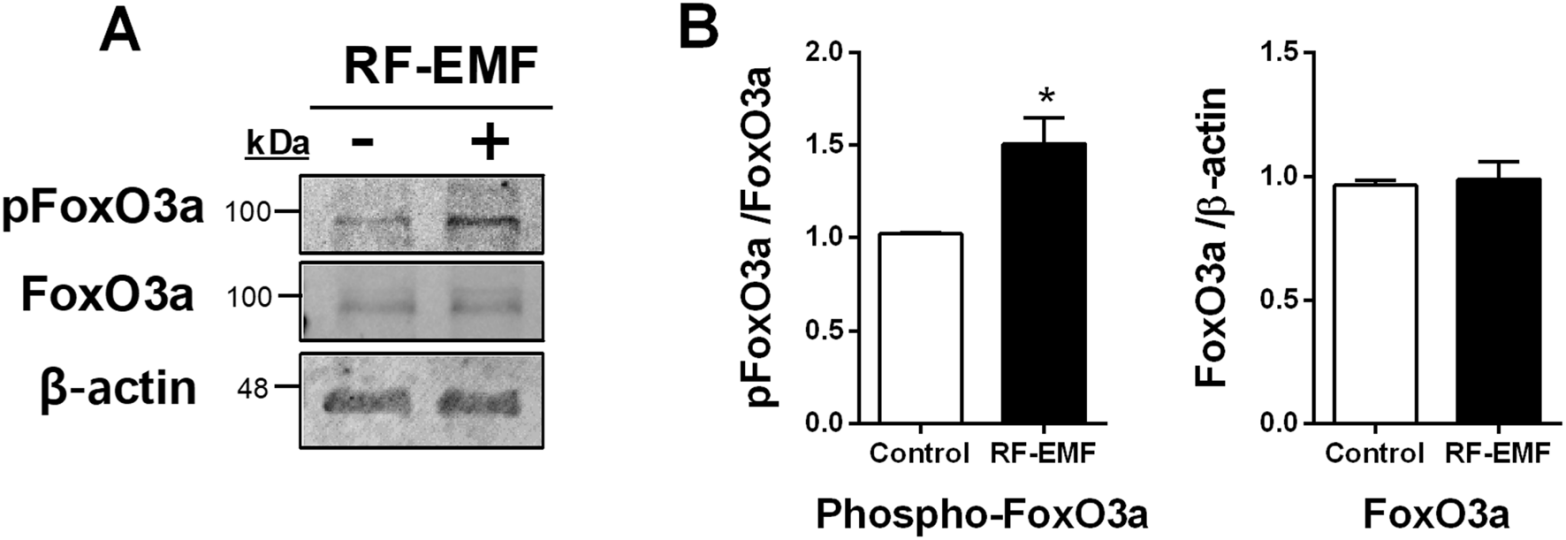
FoxO3a phosphorylation levels in HaCaT cells after radiofrequency electromagnetic field (RF-EMF) exposure. Representative immunoblot of Foxo3a and phospho-FoxO3a (Ser318/321) (**A**) in HaCaT cells after 1760 MHz RF-EMF exposure (4.0 W/kg, 2 h/day for 4 days). The expression data were normalized to β-actin levels (**B**). The bars indicate the mean ± SEM. Levels of statistical significance were evaluated using two-tailed, unpaired Student’s *t*-tests; *p < 0.05 vs. control (n = 4).

Forkhead box O3 (FoxO3) is a cellular senescent factor involved in various cellular processes such as protection from oxidative stress, metabolism, longevity, and inflammation^10^. To elucidate whether RF-EMF exposure could activate FoxO3 in human keratinocytes, cell lysates were immunoblotted with anti-FoxO3a and anti-phospho-FoxO3a (Ser 318/321) antibodies. The results showed that the FoxO3a phosphorylation level was significantly increased by approximately 1.5-fold in RF-EMF-exposed HaCaT cells. However, the total FoxO3a expression level was unaltered in human keratinocytes following this treatment (Fig. 6). Notably, FoxO3a is an adaptable player in stressed cells that functions in response to the regulation of its phosphorylation by several kinases^11^. Therefore, these data suggested that cellular stress from RF-EMF exposure would induce increased phosphorylation of FoxO3a (Ser 318/321) to prevent cellular senescence in HaCaT cells.

## Discussion

The skin is the outermost organ acting as an anatomical barrier between the internal and external environment in the body^2^. Despite its pivotal role in protecting the body, the biological effects of RF-EMF on human skin are barely known. Human exposure to 835 MHz RF with 1.6 W/kg SAR significantly decreases skin resistance, as measured using mesh-type electrodes^12^. Additionally, the expression levels of numerous proteins in human skin were found to be altered when exposed to 900 MHz RF-EMF (1.3 W/kg SAR)^3^. These studies suggest the possibility of biochemical and physiological changes in skin cells following RF-EMF exposure. In this study, we found that 1760 MHz RF-EMF (4.0 W/kg SAR for 2 h/day during 4 days) exposure stimulated intracellular ROS production in human keratinocyte HaCaT cells. Our findings strongly suggest that exposure to 1850 MHz RF-EMF induces ROS generation, leading to MMP activation and FoxO3a and ERK1/2 phosphorylation, which could induce cellular senescence in human keratinocyte HaCaT cells.

First, RF-EMF-induced intracellular ROS generation was measured using flow cytometry and fluorescence microscopy after staining with DCF-DA. Noticeably, RF-EMF exposure increased intracellular ROS levels in human keratinocytes (Fig. 1). In this experiment, the cells exposed to UVA were used as a positive control to compare ROS generation after RF-EMF exposure. These data showed that RF-EMF exposure could increase the ROS level in HaCaT cells, although RF-EMF seems to be a much weaker stimulus than UV.

According to recent statistics from Business Insiders in the UK^13^, Koreans use their mobile phones for an average of 2.1 h per day. Based on these statistics, we studied the possible biological effects of repeated exposure to 2 h per day for 4 days on HaCaT human keratinocytes. We studied the possible biological effects on cell growth by measuring cell number from day 0 to day 4, but no significant change in HaCaT human keratinocyte cell growth was observed after exposure to RF-EMF at 4 W/kg SAR for 2 h per day for 4 days (Fig. 2). In contrast, exposure to 7–10 W/kg SAR RF-EMF had a significant effect on both cell number and viability (Fig. S1). Even though there were no significant changes in cell numbers with 4 W/kg SAR RF-EMF exposure in HaCaT human keratinocytes, the intracellular ROS level was elevated and eventually led to the activation of several matrix metalloproteinases and the FoxO3a pathways, which might affect skin aging processes. We would suggest that if RF-EMF is applied at more than a 4-W/kg SAR value to HaCaT keratinocytes, the inflammatory response, as well as the apoptosis pathway, could be activated, which might induce even more rapid skin aging. Although exposure to the higher SAR values of RF-EMF should have a greater impact on the skin, this hypothesis needs to be studied by using appropriate experimental models.

Furthermore, to confirm the thermal effect of RF-EMF exposure, we examined the expression levels of heat shock proteins (HSP27, HSP70, and HSP90) that are known to be sensitive to temperature increases in cells. The results showed that there was no significant difference in all HSP proteins in HaCaT human keratinocytes after exposure to RF-EMF, as compared to the levels in control cells in our system (Fig. S2). Therefore, a possible thermal effect did not influence the cells exposed to RF-EMF in our study.

Remarkably, UV exposure to skin induces oxidative stress via ROS generation, which greatly influences the skin-aging process^4^. Fine wrinkles are formed in the skin due to reductions in collagen, elastic fibers, and hyaluronic acid^4^. Intracellular ROS generation following UV exposure to human or mouse skin upregulates the synthesis of MMPs, such as MMP1 (collagenase), MMP2 (gelatinase), MMP3 (stromelysin), MMP7 (matrilysin), MMP8 (collagenase), and MMP9 (gelatinase), which have been implicated in photoaging^4,14,15^. The major members of the matrix metalloproteinase family, MMP1 and MMP3, were significantly enhanced in HaCaT cells after RF-EMF exposure (Fig. 3A,B). MMPs can degrade extracellular matrix^4^. In addition, MMP7 expression was significantly increased in HaCaT cells following RF-EMF exposure (Fig. 3C). MMP7 greatly contributes to the degradation of elastin and proteoglycans^16^. Additionally, MMP1, 2, 3, and 9 are crucially involved in degrading the dermal extracellular matrix. Collagen is cleaved by MMP1 and is completely degraded by MMP 2, 3, and 9^4^. Moreover, MMP 2 and 9 can degrade elastic fibers^17^ and hydrolyze denatured collagen I^18^. In the gelatin zymography assay, the gelatinase activities of MMP2 and MMP9 were significantly increased in HaCaT cell culture media after RF-EMF exposure. A significantly higher proportion of active MMP2 was also observed in RF-EMF-exposed HaCaT cells (Fig. 3D). UVA irradiation can activate MMP2 and MMP9 in HaCaT human keratinocytes^19^; further, MMP2 and MMP9 are critically involved in skin photoaging after UV-induced skin damage, resulting in skin thickening and wrinkle formation^20^. These data strongly suggest that the skin-aging process, through MMP2 and MMP9 gelatinolytic activity, is induced by RF-EMF exposure in HaCaT cells.

Additionally, excessive ROS generation can induce apoptosis through extrinsic and intrinsic pathways in human keratinocytes^21,22^. Moreover, UV exposure induces ROS and causes apoptosis in human keratinocytes^22^. Apoptotic activation could eliminate genetic mutations induced by UV exposure to human keratinocytes^23^. However, RF-EMF exposure in this study did not induce apoptosis (Fig. 4). Although ROS generation in cells was significantly increased by RF-EMF exposure, the ROS level might have been insufficient to induce apoptosis in HaCaT cells.

ERK1/2 activation is mediated by the activation of MAPK cascades, which serve as the central signaling pathways and essentially govern all stimulated cellular processes^24^. Friedman et al. showed that ROS induction in Rat1 cells (rat fibroblasts) in response to RF-EMF stimulates MMP3 and then activates the ERK1/2 cascade^25^. ERK1/2 was rapidly activated in response to 875 MHz RF-EMF irradiations at various frequencies and intensities, and this activation was initially mediated by ROS produced by NADH oxidase upon RF-EMF exposure and then directly activated MMPs^25^. Noticeably, RF-EMF exposure (1760 MHz, 4.0 W/kg SAR for 2 h/d over 4 days) induced ERK1/2 phosphorylation (Fig. 5). These data agree with the hypothesis that direct MMP activation by ROS generation acts similarly to MMP1 and MMP3 activation and stimulates MMP2, MMP7, and MMP9 and then activates ERK1/2 in HaCaT cells.

Furthermore, we also examined the Cox2 expression level in HaCaT cells after RF-EMF exposure because UVB-exposed human dermal fibroblasts exhibit an inflammatory response via the induction of Cox2 expression^5^. Cox2 is an inducible enzyme for which expression is responsive to inflammatory processes. However, the level of the inflammatory mediator Cox2 was unaltered in HaCaT cells in response to RF-EMF exposure in our study.

FoxO3a belongs to the forkhead group O family of transcription factors characterized by a distinct forkhead DNA-binding domain^10^. Three other FoxO family members in humans (FoxO1, FoxO4, and FoxO6) are involved in various cellular processes, including cellular differentiation, metabolism, proliferation, and aging^10,26–29^. In particular, FoxO3 has been considered a major factor in human aging and life span^10,30,31^. Reducing FoxO3a levels could induce cellular senescence in human dermal fibroblasts^30^. Meanwhile, FoxO3a expression levels were found to be lower in old cells than in young cells, and phospho-FoxO3a (an inactivated form) levels are higher in old cells, suggesting that FoxO3a inactivation induces cellular senescence in human dermal fibroblasts^30^. Whereas FoxO3a phosphorylation was increased in human HaCaT keratinocytes after RF-EMF exposure, no significant change in FoxO3a expression was observed (Fig. 6). In addition, two conserved residue sites (Ser318 and Ser321) on FoxO3a can be phosphorylated by casein kinase 1 (CK1) in humans, leading to FoxO3a activity inhibition^32^. These data agree with our findings that RF-EMF exposure increases phospho-FoxO3a (Ser318/Ser321) and might inhibit FoxO3a activity in HaCaT cells.

In conclusion, exposure to 1760 MHz RF-EMF at 4 W/kg SAR induced intracellular ROS generation, which then stimulated MMPs (MMP1, 2, 3, 7, and 9) and activated the ERK1/2 (phospho-ERK1/2) and FoxO3a (phospho-FoxO3a) signaling pathways in HaCaT cells. Our results suggest that these changes induced by RF-EMF exposure would contribute to skin-aging processes.

## Materials and methods

### Cell culture

HaCaT human keratinocytes^33^ were generously donated by Prof. DS Kim (Chung-Ang University, College of Medicine, Seoul, Korea), originally purchased from Cell Lines Service (Eppelheim, Germany). HaCaT cells were cultured in DMEM (Gibco, Grand Island, NY) supplemented with or without 10% fetal bovine serum and 100 units of penicillin/streptomycin in a CO_2_ incubator at 37 °C. The cell number was automatically measured for 5 days before and after RF-EMF exposure using a specific program (CKX-CCSW software, Olympus, Tokyo, Japan).

### RF exposure system

The in vitro radiofrequency radiation exposure device uses a radial transmission line (RTL) exposure system that can expose multiple cells simultaneously^34,35^. Currently, a long-term evolution signal has been applied to RTL exposure systems after amplification for this study. The signal source was an uplink signal with a center frequency of 1.76 GHz, a bandwidth of 10 MHz, and quadrature phase shift keying modulation. The maximum input power was 60 W. The exposure level and time were controlled by control manipulation. The exposure signal was fed through a conical antenna with broadband characteristics. The external dimensions of the RF-EMF generator were 843 mm × 825 mm × 315 mm. The chamber was made of aluminum and served as an electromagnetic shield. The exposure system is specifically designed to control environmental conditions, including ventilation, humidity, and temperature. Gas from the incubator was circulated throughout the chamber to maintain the CO_2_ density and humidity inside the chamber. To maintain the medium temperature at 37 °C in a culture dish or flask during RF-EMF exposure, a water pump circulating water throughout the bottom of the cavity was used to control temperature. The information on this device is described in detail in a previous publication^35^.

### Cell exposure with RF-EMF exposure system

Human keratinocyte HaCaT cells were cultured in DMEM, supplemented with 10% (v/v) fetal bovine serum (Gibco, Grand Island, NY) and 100 units of penicillin/streptomycin (Gibco, Grand Island, NY), in a 5% CO_2_ incubator at 37 °C. Cells were exposed to RF-EMF using a 1760 MHz RF-EMF RTL exposure system with a SAR value of 4.0 W/kg (5% SD, based on 5 different SAR measurement locations within the petri-dish) for 2 h daily for 4 days. During the RF-EMF exposure, the temperature of the culture media in the chamber was maintained at 37 °C by circulating water within the cavity, and 5% CO_2_ was maintained in the RF-EMF generator. The sham-treated cells were kept under identical environmental conditions as the RF-EMF-exposed cells, except for RF-EMF exposure.

### Cell proliferation assay

Cell proliferation was estimated using a colorimetric method based on water-soluble tetrazolium salts (WST-1, Abfrontier, Seoul, Korea) according to the manufacturer’s instructions. HaCaT cells were grown and seeded in 24-well plates at a density of 3 × 10^4^ cells in 400 μL of medium per well. Briefly, 40 μL of the reagent was added to each well, and the plates were incubated at 37 °C for 3 h. The samples were transferred to a 96-well microplate. The proliferation rate was determined by measuring the absorbance of each well at a wavelength of 450 nm using a 96-well microplate spectrophotometer (Multiskan GO; Thermo Scientific, Waltham, MA).

### Measurement of intracellular ROS generation

Intracellular ROS production was detected by staining cells with the fluorescent probe DCF-DA purchased from Sigma-Aldrich Co (#D6883, St. Louis, MO). This dye is a stable compound that readily diffuses into cells and is hydrolyzed by intracellular esterase to yield DCFH, which is trapped within cells. HaCaT cells were incubated in 24-well plates at a density of 1 × 10^5^ cells per well for 24 h before UVA irradiation. Following 6 h incubation after UVA irradiation, to produce cellular fluorescence images, the cells were incubated with 10 μM DCF-DA at 37 °C for 30 min in the dark and then washed twice with PBS. The fluorescence images were obtained using a confocal microscope (FV-3000, Olympus, Tokyo, Japan). For flow cytometric analysis, the cells were not washed after staining with 10 μM DCF-DA. Cellular ROS levels were then analyzed by flow cytometry using a BD Accuri C6 flow cytometer with C6 Software (BD Biosciences, San Jose, CA).

### Immunoblotting analysis

HaCaT cells were lysed with RIPA buffer (Thermo Scientific, Rockford, IL) supplemented with a protease and phosphate inhibitor cocktail (Thermo Scientific, Rockford, IL). Whole cell lysates were sonicated briefly. Protein concentrations were measured using a protein assay kit (Bio-Rad, Hercules, CA) and total proteins (20–50 μg) were separated by 10% sodium dodecyl sulfate–polyacrylamide gel electrophoresis (SDS-PAGE) and transferred to a polyvinylidene difluoride transfer membrane (Bio-Rad, Hercules, CA). MMP1, MMP3, Bcl2, Bax, FoxO3a, phospho-FoxO3a, ERK1/2, phospho-ERK1/2, Cox2, and α-tubulin or β-actin were detected in the membranes using anti-MMP1 (1:1000, Abcam, Cambridge, UK #ab137332), anti-MMP3 (1:1000, Flarebio, College Park, MD #CSB-PA07449A0Rb), anti-MMP7 (1:1000, Flarebio #CSB-PA014677EA01Ra), anti-Bcl2 (1:500, Cell Signaling Technology, Danvers, MA #2876), anti-Bax (1:500, Cell Signaling Technology #14796), anti-FoxO3a (1:500, Abcam #ab109629), anti-phospho-FoxO3a (Ser318/321; 1:500, Cell Signaling Technology #13129P), anti-p44/42 MAPK (1:500, Cell Signaling Technology #9102), anti-phospho-p44/42 MAPK (Thr202/Tyr204; 1:500, Cell Signaling Technology #9101L), anti-Cox2 (1:500, Abcam #ab15191), anti-HSP27 (Santa Cruz, Dallas, TX #sc-13132), anti-α-tubulin (1:3000, Santa Cruz #sc-23948), and anti-β-actin (1:3000, Sigma-Aldrich #A5441) antibodies. The protein bands were visualized using an Odyssey infrared imaging system (Li-Cor Biosciences, Lincoln, NE). The intensity of each band was quantified and normalized using α-tubulin or β-actin as an internal loading control.

### Gelatin zymography

The amounts of MMP2 and MMP9 secreted into the culture media were measured by gelatin zymography^18^. The cells were collected after 24 h of incubation following RF-EMF exposure (1760 MHz, 4.0 W/kg for 2 h daily for 4 days). For the positive control, the cells were treated with UVA for 45 min. The collected cell media (supernatants) were then concentrated using a centrifugal concentrator (Vivaspin 500, Sartorius Stedim Lab Ltd, Stonehouse, UK). Each sample was mixed with non-reducing sample buffer and loaded onto the SDS-gel without prior heating. The samples were separated by SDS-PAGE on a 7.5% polyacrylamide gel containing 0.4% gelatin at 150 V for 2 h in a Bio-Rad Mini PROTEAN 3 Cell electrophoretic apparatus (Bio-Rad, Hercules, CA). After electrophoresis, the gel was thoroughly washed twice using 2.5% triton X-100 and then incubated in 20 mL reaction buffer (50 mM tris–HCl, pH 7.5, containing 5 mM CaCl_2_, 1 µM ZnCl_2_, and 2.5% triton X-100) at 37 °C for 24 h and finally stained with Coomassie brilliant blue R-250^36^. MMP2 and MMP9 were detected at 72 and 92 kDa, respectively, as clear zones against the dark background. Relative band densities were analyzed using an Odyssey infrared imaging system (Li-Cor Biosciences, Lincoln, NE). Each gelatinolytic activities were normalized by using a total amount of loaded proteins.

### Statistical analysis

All data are presented as the mean ± SEM. The n-value represents the number of independent samples used in the experiments. The significance for all pairwise comparisons of interest was assessed using the two-tailed Student’s *t*-test, and *p* < 0.05 was considered statistically significant. All experiments were independently performed with at least three different samples. GraphPad Prism 4 software (GraphPad Software, La Jolla, CA) was used for statistical analysis.

## Supplementary Information

Supplementary Information
