# Supplementary material for: Activation of matrix metalloproteinases and FoxO3a in HaCaT keratinocytes by radiofrequency electromagnetic field exposure
Source: Sci Rep. 2021 Apr 7;11:7680. doi: 10.1038/s41598-021-87263-2 (PMC8027011; doi:10.1038/s41598-021-87263-2)

# Activation of Matrix Metalloproteinases and FoxO3a in HaCaT Keratinocytes by Radiofrequency Electromagnetic Field Exposure

Ju Hwan Kim[1], Dong-Jun Kang[1], Jun-Sang Bae [2], Jai Hyuen Lee[3], Sangbong Jeon[4], Hyung-Do Choi[4], Nam Kim[5], Hyung-Gun Kim[1], and Hak Rim Kim[1]*

[1] Department of Pharmacology, College of Medicine, Dankook University, Cheonan, Chungnam, South Korea, 31116

[2] Medical Laser Research Center, Dankook University, Cheonan, Chungnam,

[3] Department of Nuclear Medicine, College of Medicine, Dankook University

[4] Radio and Broadcasting Technology Laboratory, ETRI, Daejeon, South Korea, 34129

[5] School of Electrical and Computer Engineering, Chungbuk National University, Cheongju, Chungbuk, South Korea, 28644

Running title: Skin aging in HaCaT cell by RF-EMF

*Correspondence: Hak Rim Kim, Ph.D
Department of Pharmacology
College of Medicine, Dankook University
119 Dandaero, Cheonan,
Chungnam, 31116 ROK
Tel: +82-41-550-3935

Email: hrkim@dankook.ac.kr

# Supplementary information

**Cell number and viability with different SAR values in HaCaT keratinocyte cells**.

We studied the possible biological effects on cell growth by measuring cell number from day 0 to day 4, but no significant change in HaCaT human keratinocyte cell growth was observed after exposure to RF-EMF at 4 W/kg SAR for 2 h per day for 4 d. In contrast, exposure to 7–10 W/kg SAR RF-EMF had a significant effect on both cell number and viability (Fig S1).

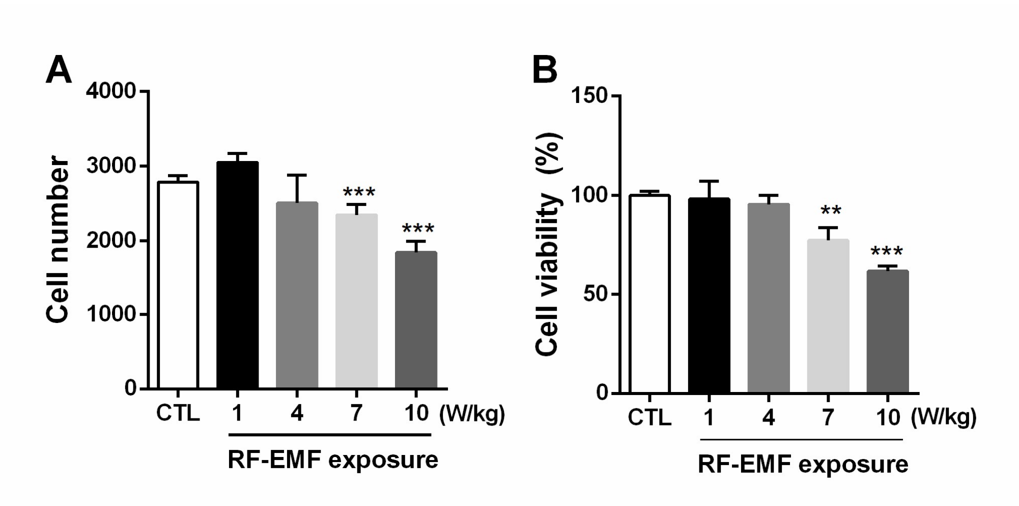


**Fig S1. RF-EMF exposure with different SAR values in HaCaT keratinocyte cells**. HaCaT cells were exposed to 1760 MHz RF-EMF with different SAR value (0, 1, 4, 7, 10 W/kg) 2h/day for 4 days. **A.** The number of cells was counted using CKX-CCSW software, which provides a record of quantitative data automatically. CTL; control. **B.** Cell viability was measured on day 5 using tetrazolium salts (WST-1) Cell Proliferation Assay. The data indicate the mean ± SEM. Statistically significant level was evaluated using two-tailed, unpaired Student's *t*-test. $^{**}p < 0.01$, $^{***}p < 0.001$ vs. control (n = 3).

**Expression level of HSPs in human HaCaT cell after RF-EMF exposure.**

To confirm the thermal effect of RF-EMF exposure, we examined the expression levels of heat shock proteins (HSP27, HSP70, and HSP90). The results showed that there was no significant difference in all HSP proteins in HaCaT human keratinocytes after exposure to 1760 MHz RF-EMF at 4.0W/kg SAR for 2 h daily for 4 days, as compared to the levels in control cells in our system (Fig S2). Therefore, a possible thermal effect did not influence the cells exposed to RF-EMF in this study.

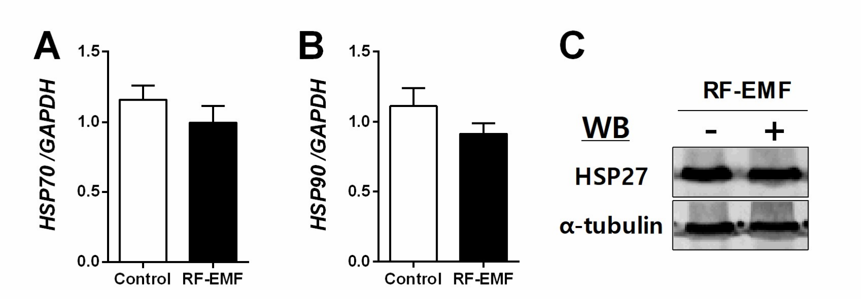


**Figure S2. Expression level of HSPs in human HaCaT cell after RF-EMF exposure.** Human keratinocyte HaCaT cells were exposed to 1760 MHz RF-EMF at 4.0W/kg SAR for 2 h daily for 4 days. Quantification of HSP70 (**A**), and HSP90 (**B**) mRNA transcripts by qRT-PCR. Total protein extracted from HaCaT cell after RF-EMF exposure was immunoblotted with antibody against HSP27 (**C**) (n=3).

# Original images of full-length blots

## Figure 3A

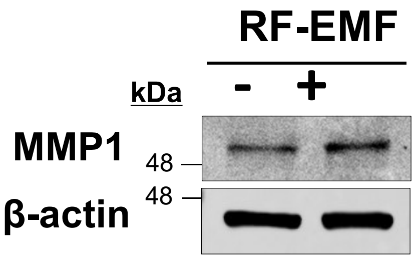


MMP1

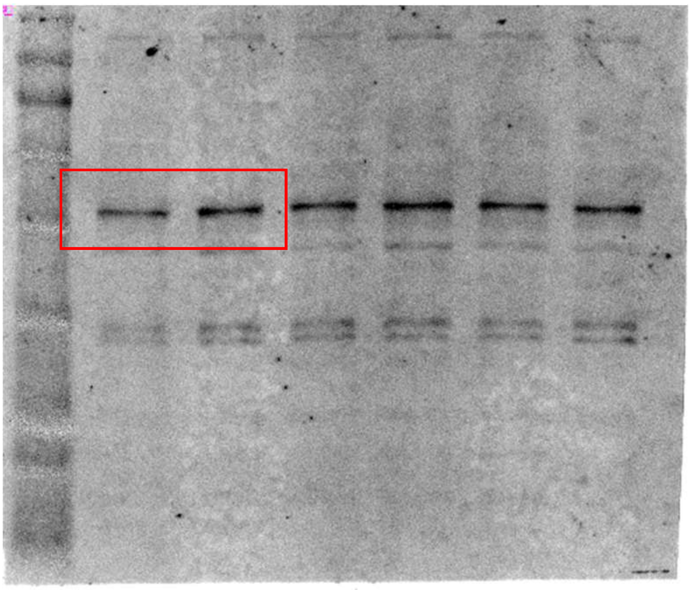

β-actin

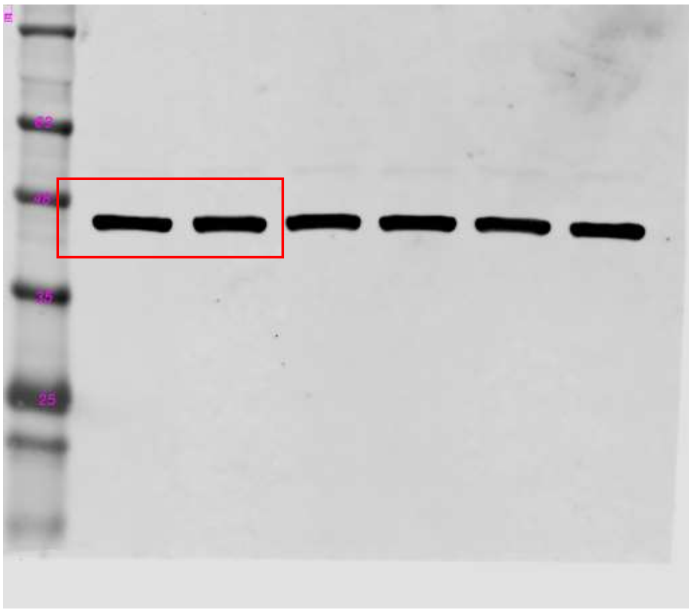

# Figure 3B

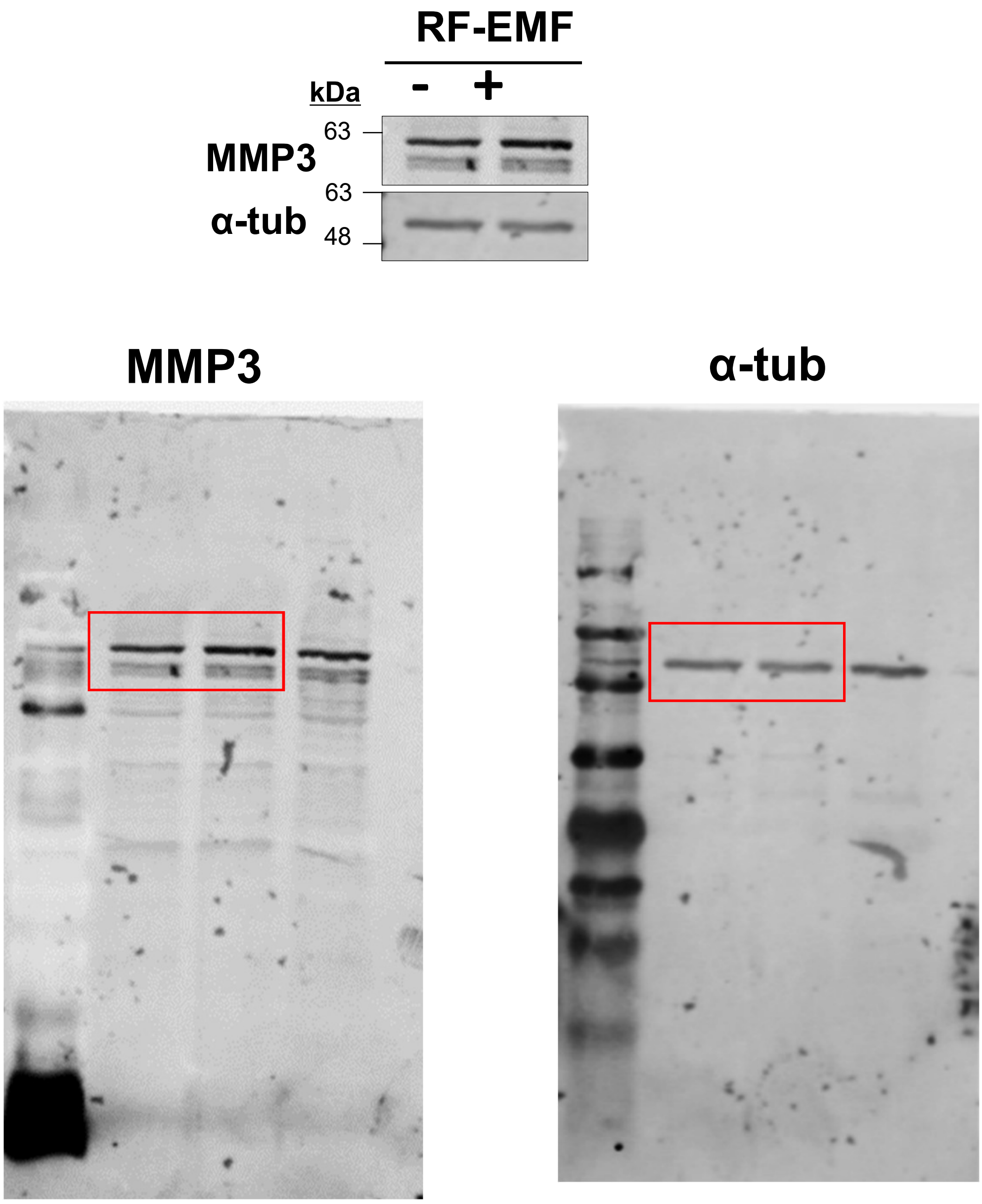

# Figure 3C

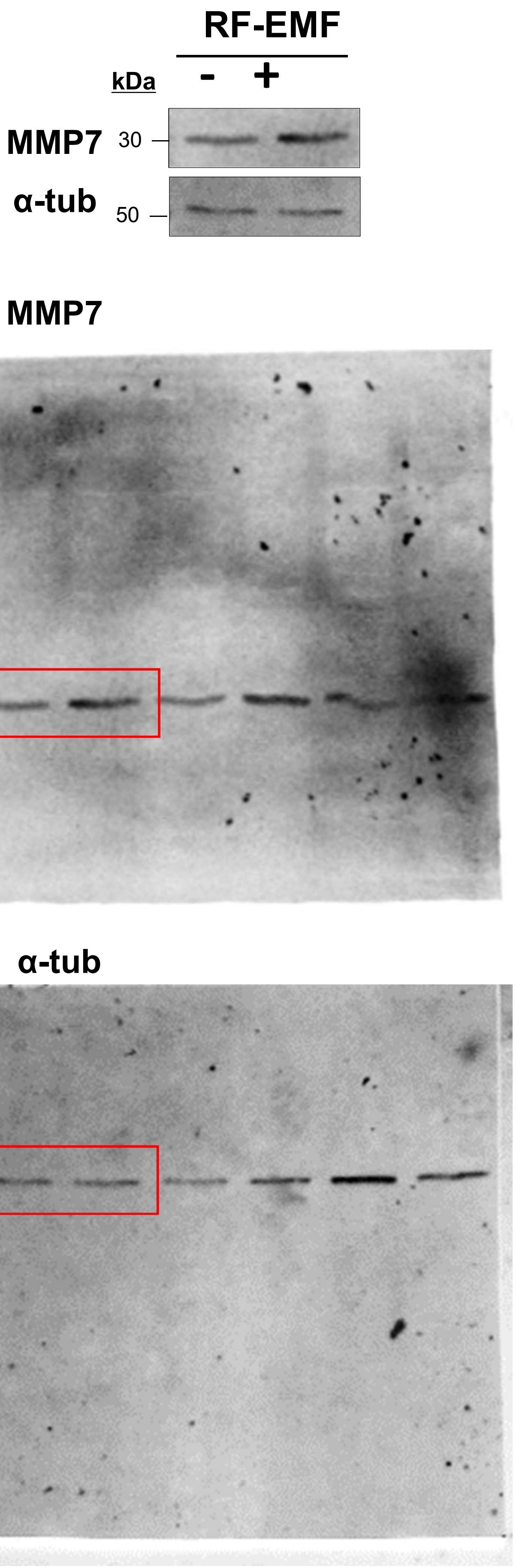

# Figure 3D

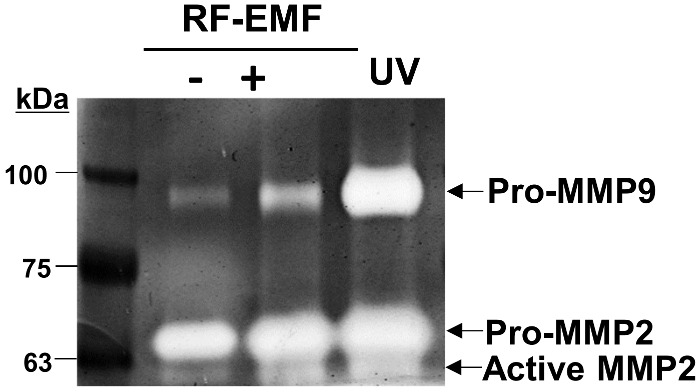


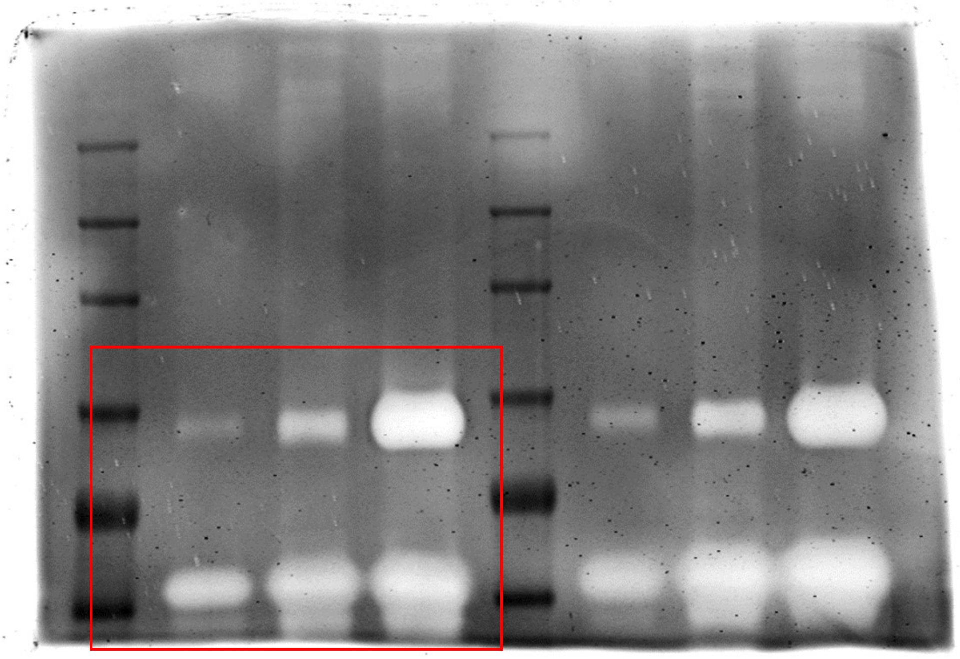

# Figure 4

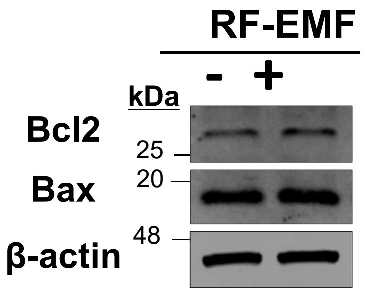


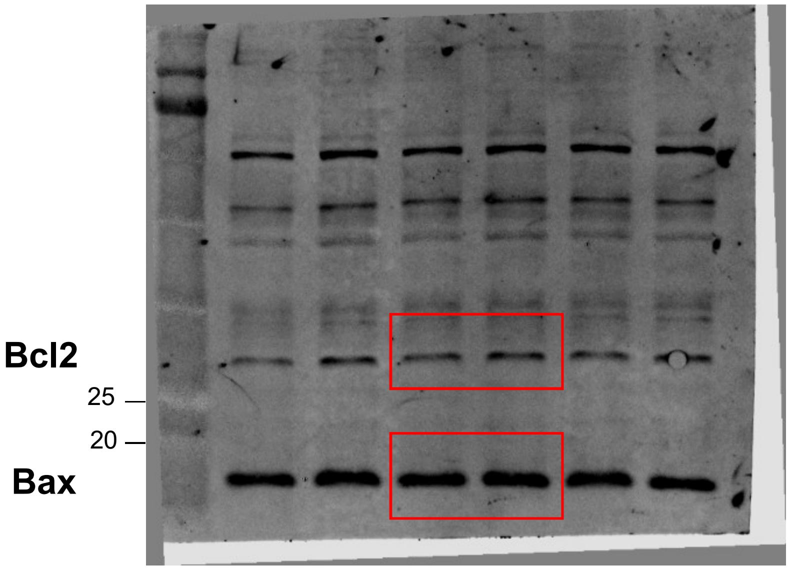


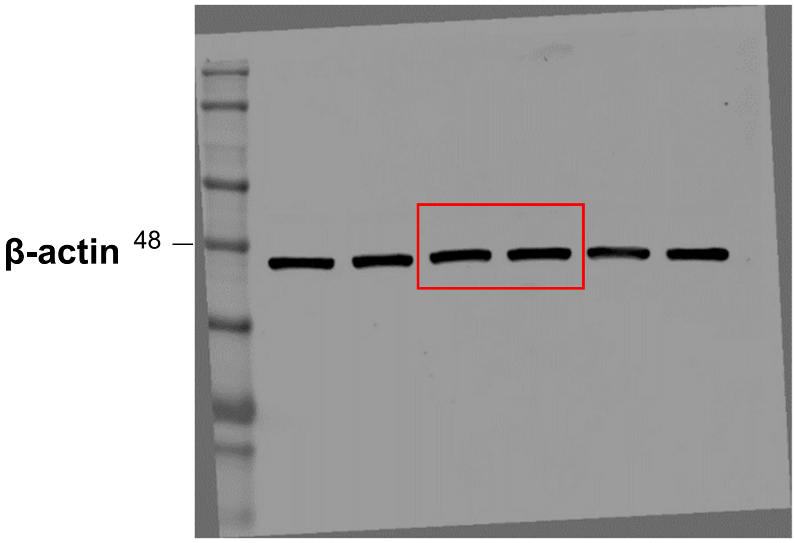

# Figure 5A

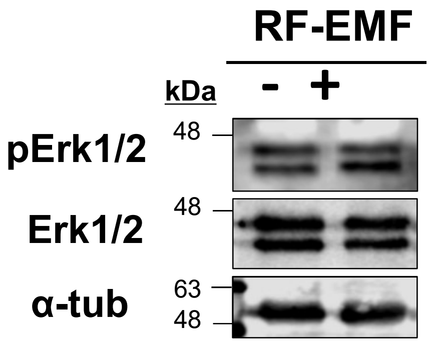


pErk1/2

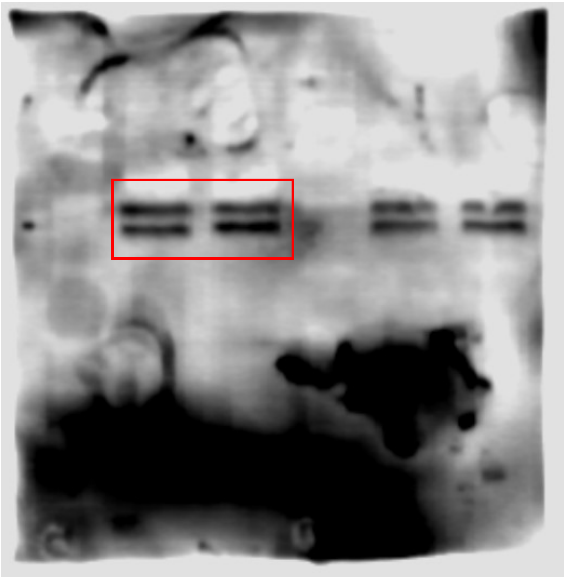

Erk1/2

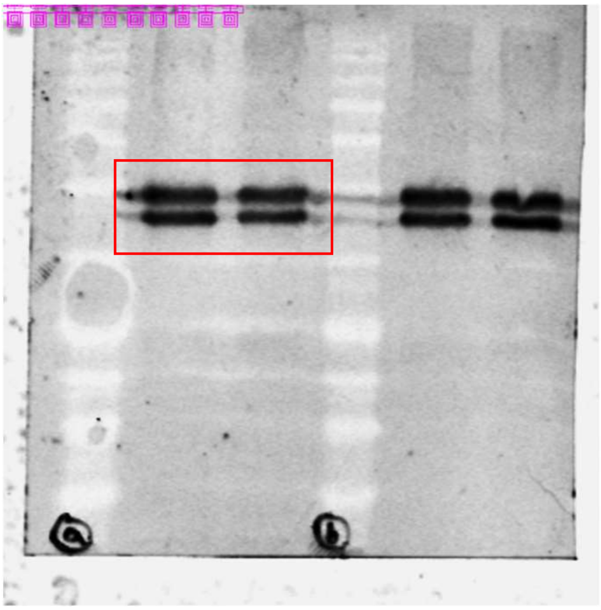

α-tub

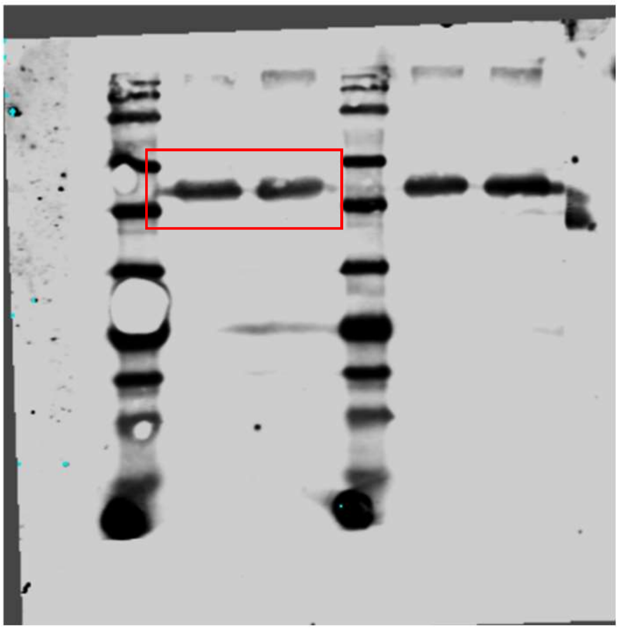

# Figure 5B

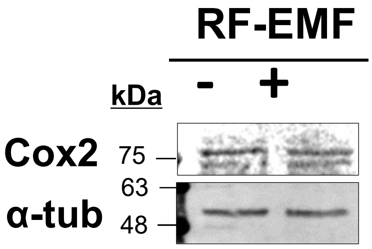


Cox2

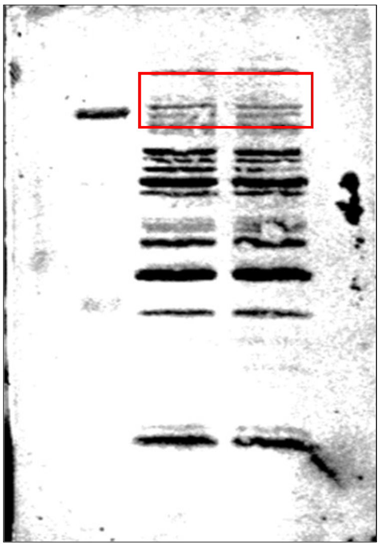

α-tub

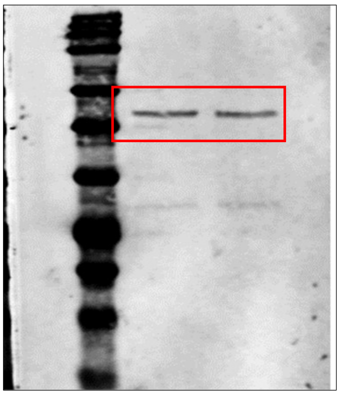

# Figure 6

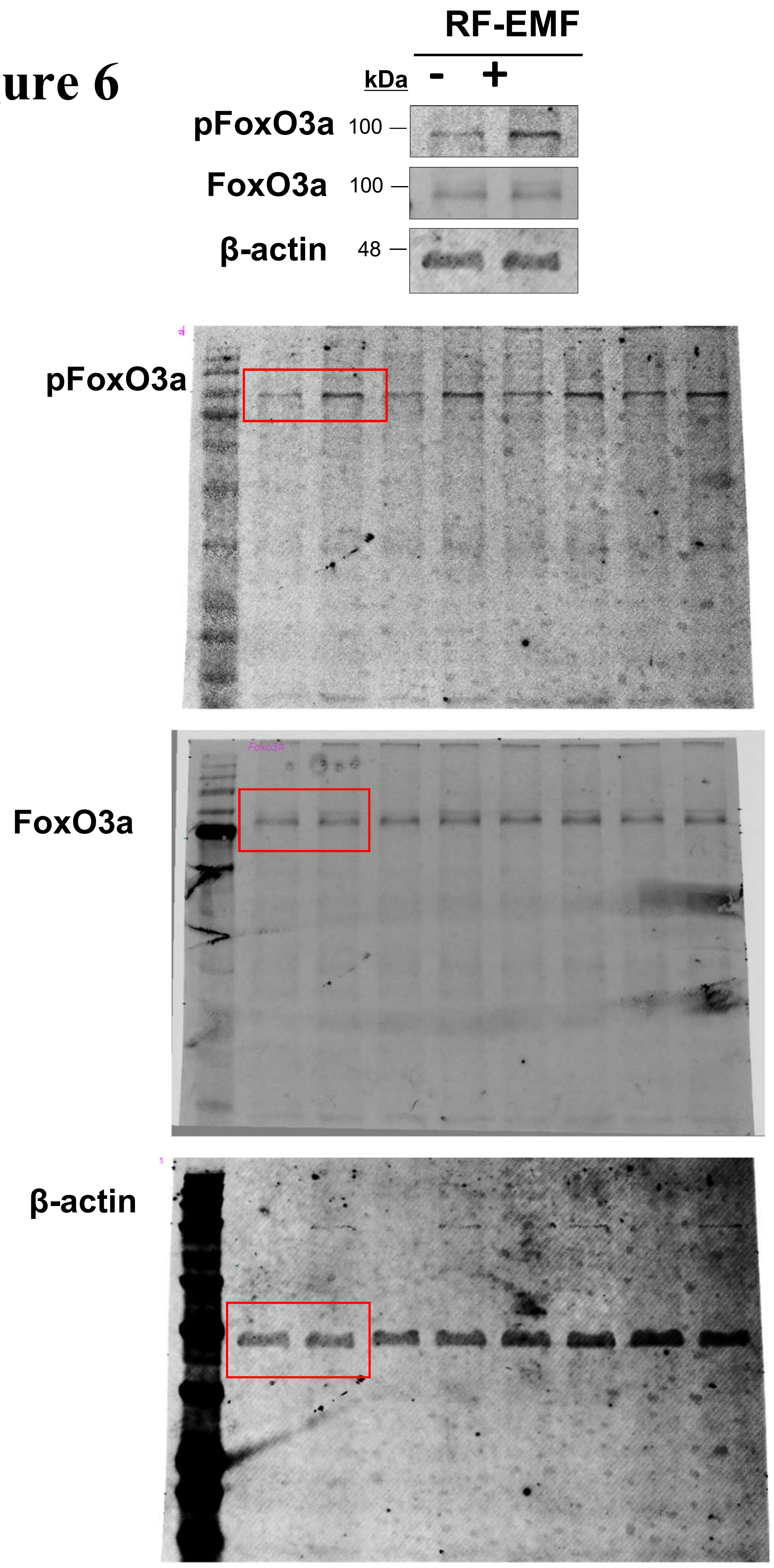
RF-EMF
kDa
-
+
pFoxO3a
100
FoxO3a
100
β-actin
48
pFoxO3a
FoxO3a
β-actin

# Figure S2

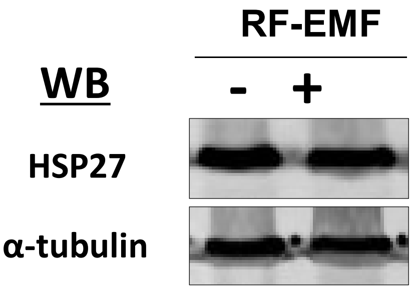


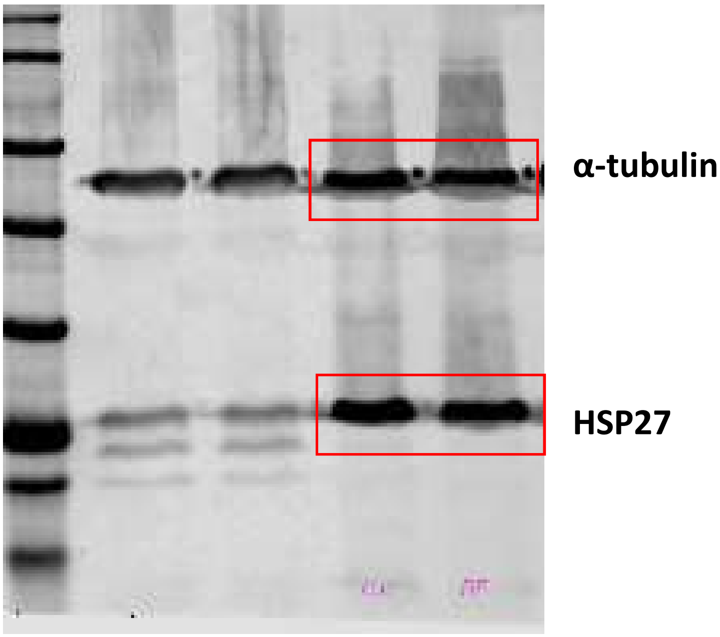

Supplement: Supplementary file 1 — Supplementary Information [file 41598_2021_87263_MOESM1_ESM.pdf]
